# Supplementary material for: Cortical-striatal brain network distinguishes deepfake from real speaker identity
Source: Commun Biol. 2024 Jun 11;7:711. doi: 10.1038/s42003-024-06372-6 (PMC11166919; doi:10.1038/s42003-024-06372-6)
Supplement: Supplementary file 2 — Supplementary Information [file 42003_2024_6372_MOESM2_ESM.pdf]

SUPPLEMENTARY INFORMATION

Cortical-striatal brain network distinguishes deepfake from real speaker identity

Claudia Roswandowitz[1,2,3], Thayabaran Kathiresan[2], Elisa Pellegrino[2], Volker Dellwo[2], Sascha Frühholz[1,3,4]

*Correspondence to: claudia.roswandowitz@uzh.ch

This PDF file includes:

Supplementary Methods

Supplementary Figures 1 to 4

Supplementary Tables 1 to 14

SUPPLEMENTARY METHODS

Addition information on natural speaker recordings

All recordings were taken in a sound-shielded booth under the same lighting condition in front of a black background. Speakers were visible from the shoulders to the top hairline with a neutral expression. No face contained salient visual features such as beards, piercings, or glasses. High-quality auditory recordings were taken by using a Neumann STH-100 transducer microphone (sampling rate 32kHz; 16kbit quantization) and Praat software. Sentences were prompted on a computer screen inside the booth through a customized Praat-Plug-in (Presenter Pro 2). In parallel, high-quality video recordings were taken with a digital video camera (Sony HDR-SR12E). Post-processing of the auditory stimuli was done with customized Praat scripts and stimuli were normalized for intensity (68dB). For the audio-visual stimuli, audio and visual tracks were aligned and cut with the open-source video editor Shotcut (https://www.shotcut.org). High-quality auditory stimuli were normalized in intensity (68dB, Praat) and merged with the visual track. Final audio-visual stimuli were exported into AVI format.

Additional information on the synthesis of deepfake voice identities and speech utterances

The VC technique consists of two major processing steps referred to as "training" and "conversion". Before the training, we down-sampled the speech signals from 32kHz to 22kHz to match the requirements of the SPROCKET tool. During the training step, acoustic voice features, including the voice fundamental frequency (F0), aperiodicity, and mel-cepstrum, were parameterized from the spectral envelope from the speech signals of both the source and target speakers. Because the source and target speakers utter the sentences with different temporal duration, a frame-by-frame iterative alignment process based on dynamic time warping (DTW) was performed. The time-aligned features are used to model a joint probability density function based on the GMM. We tuned the hyperparameters of the GMM

conversion model, and we observed the best results by using a 32-mixture GMM with full covariance. In the conversion step, the F0 and the mel-cepstrum of the source speaker are converted into those of the target speaker by using the trained GMM. Other acoustic features, such as aperiodicity, speaking rate, temporal structure of the F0 trajectory, and power trajectory, are not converted. Therefore, the converted speech retains the temporal aspects of the source speaker.

SUPPLEMENTARY FIGURES

Supplementary Figure 1. Experimental design and accuracies of the speaker familiarization task. (a) Participants (n=30) were familiarized with the natural vocal identity of the four male speakers in an audio-visual familiarization task. Participants learned the natural voices together with the synchronized dynamic face and the speaker's name. In the testing phase, participants listened to a voice and selected in a four-alternative forced-choice task the corresponding face/name matching the speaker's voice. The familiarization task included seven learning and seven testing phases. We have obtained consent of the speakers depicted for the publication of identifiable images. **(b)** Accuracy of the familiarization task. Black line indicates mean accuracy of 96.83% with a range of 87.50-100%. Individual performance is plotted for the participants (n=27) with above 80% correct in the last two testing phases, which we defined as the critical learning threshold to complete the main fMRI matching task. Circles indicate individual performances and the horizontal line the mean percent correct over all participants.

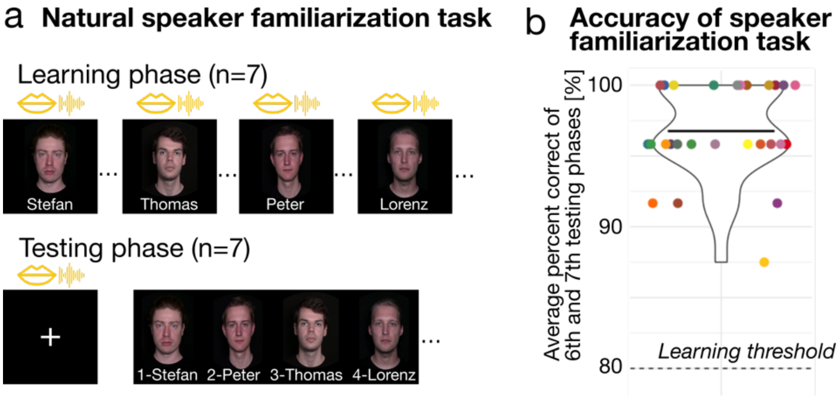

Supplementary Figure 2. Individual task performance in the natural identity and natural speech matching task. Circles indicate individual performances of the 25 participants. Blue lines (n=6) indicate participants with superior performance in the natural identity as compared to the natural speech matching task, red lines indicate participants with superior performance in the natural speech as compared to the natural identity matching task. Horizontal black lines show mean percent correct over all participants for the respective task.

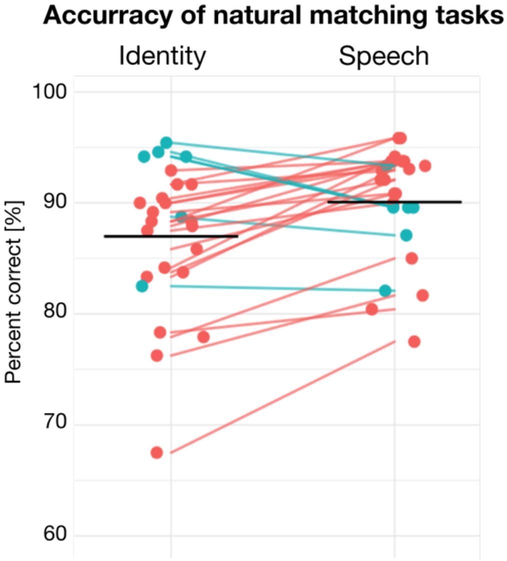

Supplementary Figure 3. Local brain responses for the identity and speech-matching task. (a-b) Neural activity patterns for contrasting natural and deepfake conditions during the identity-matching task (second-level group t-maps, p<.005 corrected at the cluster level k>47). See SupplementaryTable 4 for statistical peak coordinates. **(c)** Neural activity patterns for contrasting natural and deepfake conditions during the speech-matching task (second-level group t-maps, p<.005 corrected at the cluster level k>47). No brain region was significantly active for the contrast $SPEECH_{nat}$>$SPEECH_{df}$. See Supplementary Table 5 for statistical peak coordinates. White dashed line indicates voice-sensitive regions evoked by the functional voice localizer scan (second-level group t-maps, p<.005 corrected at the cluster level k>47).

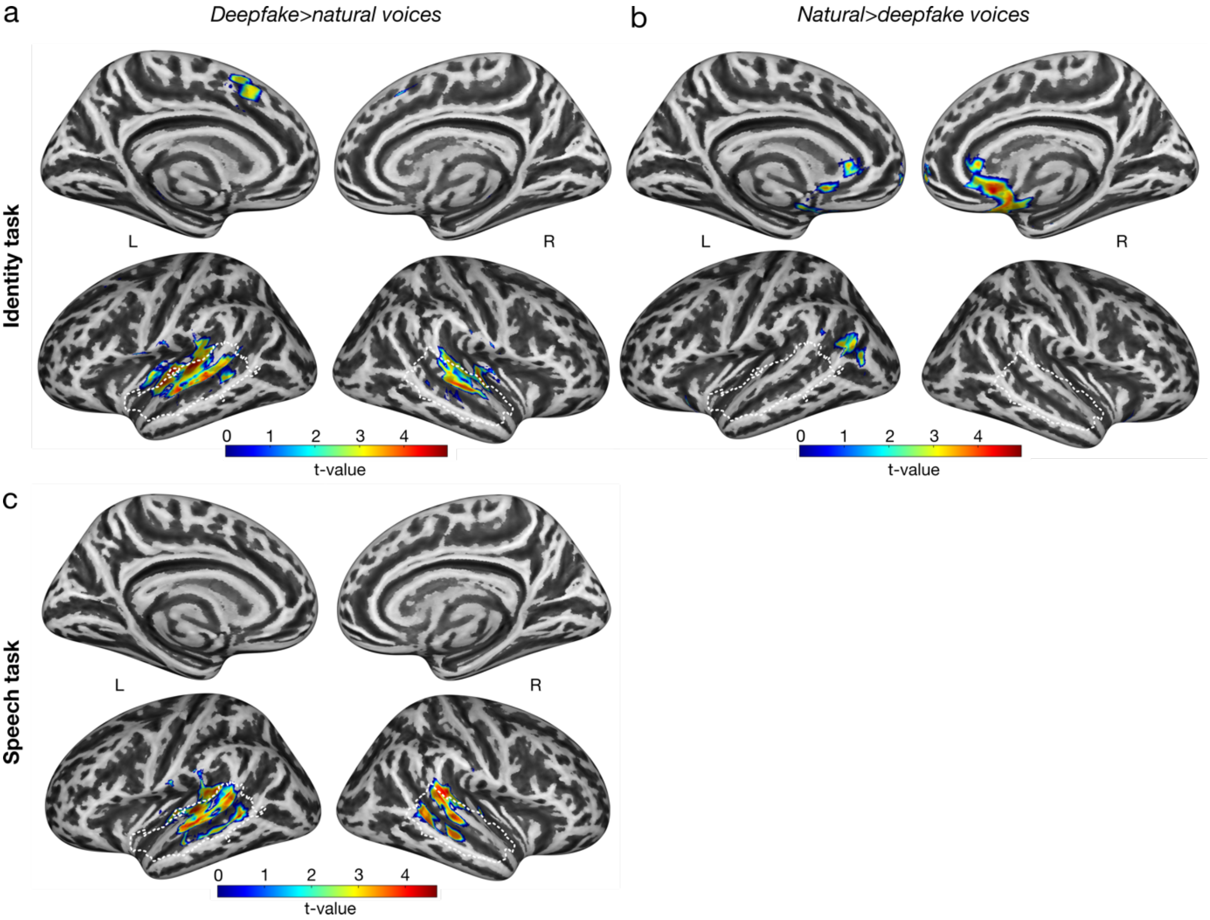

Supplementary Figure 4. Social ratings on the natural and deepfake voices. Plotted are the absolute counts of participants' (n=25) ratings on (**a**) voice naturalness, (**b**) voice likability, and (**c**) voice trustworthiness.

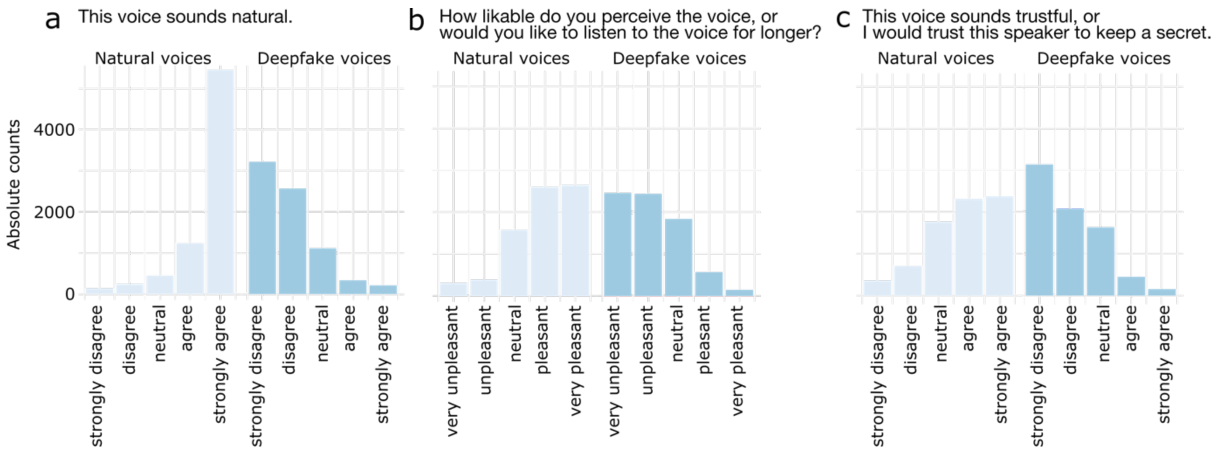

SUPPLEMENTARY TABLES

Supplementary Table 1. The representation of acoustic voice-identity parameters in natural and deepfake voices and the effect of sound condition. Mean and standard deviation of the acoustic parameters encoding voice identity measured in the set of natural and deepfake sounds presented in the fMRI matching task. Model outputs for the linear mixed model (LMM), assessing effects between the natural and deepfake voices for the identity encoding voice features. We ran LMMs on standardized acoustic values to increase model output comparisons. We consider *p*-values significant at $p<.007$, Bonferroni corrected for the number of acoustic parameters (n=7).

	**Natural voices (n=332)**	**Deepfake voices (n=332)**	**LMM (*n* observations=664)** Fixed effect: sound condition			Random effects Speaker (n=4)	Random effects Sentence (n=83)	Explained model variance
	Mean (SD)	Mean (SD)	Estimate	CI	*p* value	Variance	Variance	Conditional R^2
**Mean F0 [Hz]**	107.83 (25.81)	106.34 (17.11)	-0.17	-0.17;0.03	.174	0.76	0.01	0.652
**SD F0 [Hz]**	16.97 (26.87)	14.56 (15.77)	-0.11	-0.26;0.04	.145	0.02	0.06	0.079
**Jitter [%]**	0.015 (0.006)	0.021 (0.05)	0.88	0.76;0.99	<.001	0.13	0.16	0.465
**Mean Harmonicity [dB]**	8.67 (2.94)	6.26 (1.82)	-0.89	-0.97;-0.80	<.001	0.22	0.31	0.684
**SD Harmonicity [dB]**	5.60 (1.32)	5.66 (1.03)	0.05	-0.06;0.17	.371	0.20	0.28	0.460
**Formant Dispersion [Hz]**	964.81 (49.60)	994.57 (36.35)	0.65	0.57;0.72	<.001	0.21	0.49	0.760
**Speech rate [syl/s]**	3.81 (0.85)	3.33 (0.65)	-0.60	-0.69;-0.51	<.001	0.07	0.50	0.649

Supplementary Table 2. Behavioral performance on the identity and speech-matching task. Model outputs for the linear mixed model, assessing task and sound-condition effects and their interaction for the behavioral matching responses obtained by the fMRI matching task. For factors, reference level is shown in brackets. Post hoc analysis on the significant interaction was done with the R function *emmeans*.

**Factors predicting task performance**	**Estimate**	**CI**	***p* value**
Task condition [speech]	3.09	0.43;5.74	.023
Sound condition [deepfake]	-18.05	-20.70;-15.39	<.001
Task × sound condition	16.72	12.96;20.47	<.001

$N_{participants}$=25, $N_{observations}$=100, random effect: VP variance=19.04, Conditional R^2 =0.803

**Post hoc analysis**	**Estimate**	**CI**	***p* value**
Identity task<br>Natural vs deepfake voices	18.05	15.35;20.75	<.0001
Speech task<br>Natural vs deepfake voices	1.33	-1.37;4.03	.33

Supplementary Table 3. Signal detection analysis on the responses of the identity and speech-matching task. *Hit*: same target/test sound and same response, correct rejection *CR*: different target/test sound and different response, *Miss*: same target/test sound and different response, false alarm *FA*: different target/test sound and same response; n=25.

	**Correct responses**		**Incorrect responses**	
	**Hit**	**CR**	**Miss**	**FA**
**ID_{nat}**	89.22	87.96	10.78	12.04
**ID_{df}**	71.36	70.14	28.64	29.86
**$SPEECH_{nat}$**	91.79	91.21	8.21	8.79
**$SPEECH_{df}$**	90.77	89.61	9.23	10.39

Supplementary Table 4. Post-error slowing analysis (PES) of the identity and speech matching task. a) PES per task and sound condition, i.e. reaction time of correct post- minus correct pre-error trials, see "robust" post-error slowing (*1*). Positive values indicate slower reaction times after error trials. **b)** Model outputs for the linear mixed model, assessing task and sound-condition effects and their interaction for the PES. For factors, reference level is shown in brackets. **c)** Outcome of a one Sample t-test testing a PES effect in the deepfake identity task, which was associated with the highest number of errors; n=25.

**a)**	**Post-error slowing in ms**		
		**Mean**	**SD**
Natural identity task		329.05	3693.876
Deepfake identity task		40.51	3374.240
Natural speech task		646.54	2597.362
Deepfake speech task		4.49	3008.720
**b) Factors predicting PES**	**Estimate**	**CI**	***p* value**
Task condition [speech]	317.50	-267.54;902.53	.287
Sound condition [deepfake]	-288.54	-745.91;168.83	.216
Task × sound condition	-353.52	-1131.99;424.96	.373
$N_{observations}$=1257, Conditional R^2 =0.002			
**c) One Sample t-test (mean greater than 0)**	**t**	**df**	***p* value**
Deepfake identity task	0.269	501	.394

Supplementary Table 5. Peak co-ordinates of univariate BOLD analysis contrasting neural response to natural and deepfake identity. Significance threshold was set at p<.005 corrected at the cluster level k>47, n=25.

Region	Cluster size	Z value	MNI coordinates		
			x	y	z
Contrast: ID_{nat}>ID_{df}					
**R nucleus accumbens**	**300**	**5.00**	**10**	**12**	**-6**
R inferior frontal gyrus, orbitalis		3.50	18	12	-14
R middle orbital gyrus		2.99	6	24	-6
**L anterior cingulate gyrus**	**61**	**3.88**	**-2**	**26**	**2**
L anterior cingulate gyrus		3.03	-10	32	0
**L superior orbital gyrus**	**134**	**3.59**	**-16**	**14**	**-10**
L nucleus accumbens		3.35	-6	14	-6
L olfactory cortex		2.94	-16	4	-12
**R mid orbital gyrus**	**55**	**3.40**	**6**	**58**	**-8**
L mid orbital gyrus		3.00	-2	60	-8
**L middle temporal gyrus**	**49**	**3.13**	**-48**	**-60**	**18**
L middle temporal gyrus		2.80	-54	-66	14
Contrast: (ID_{nat}>ID_{syn})>($SPEECH_{nat}$>$SPEECH_{df}$)					
**R nucleus accumbens**	**82**	**4.06**	**10**	**12**	**-6**
R inferior frontal gyrus, orbitalis		2.75	18	14	-14
Contrast: ID_{df}>ID_{nat}					
**L insula**	**1196**	**4.29**	**-44**	**-26**	**0**
L superior temporal gyrus		4.14	-54	-26	4
L superior temporal gyrus		4.05	-48	-10	-6
**R superior temporal gyrus**	**512**	**4.08**	**50**	**-16**	**-4**
R superior temporal gyrus		3.86	52	-6	-6
R superior temporal gyrus		3.35	62	-20	2
**L superior frontal gyrus**	**140**	**3.47**	**-12**	**14**	**50**
L white matter		3.24	-12	20	44
Contrast: (ID_{df}>ID_{nat})>($SPEECH_{df}$>$SPEECH_{nat}$)					
**L white matter**	**50**	**3.87**	**-24**	**-32**	**22**
L parietal operculum		2.95	-34	-38	24

Supplementary Table 6. Peak co-ordinates of univariate BOLD analysis contrasting neural response to natural and deepfake speech. Significance threshold was set at *p*<.005 corrected at the cluster level k>47, n=25.

Region	Cluster size	Z value	MNI coordinates		
			x	y	z
Contrast: $SPEECH_{nat}$>$SPEECH_{df}$					
**L white matter**	**90**	**4.24**	**-24**	**-32**	**22**
L gray matter		3.36	-22	-40	24
Contrast: $SPEECH_{df}$>$SPEECH_{nat}$					
**R superior temporal gyrus, Te3**	**476**	**4.04**	**64**	**-30**	**8**
R superior temporal gyrus		3.79	54	-22	-2
R middle temporal gyrus		3.66	64	-38	6
**L middle temporal gyrus**	**826**	**4.26**	**-64**	**-44**	**6**
L superior temporal gyrus		3.87	-56	-18	2
L middle temporal gyrus		3.66	-54	-38	10

Supplementary Table 7. Peak co-ordinates for significant brain regions of the functional network analysis. The table reports functional connectivity during natural and deepfake identity matching (p<.005 corrected at cluster level k>47, n=25). Seed regions are based on the univariate contrast analysis (Figure 2a, Supplementary Table 5). For completeness, we report functional connectivity with the left nucleus accumbens in gray font.

Region	Cluster size	Z value	MNI coordinates		
			x	y	z
Contrast: ID_{nat}>baseline					
SEED R nucleus accumbens					
**L supplementary motor cortex**	**912**	**4.49**	**-12**	**-4**	**54**
R middle cingulate cortex		4.36	8	4	42
L middle cingulate cortex		4.05	-8	4	46
**L thalamus**	**292**	**4.38**	**-8**	**-20**	**-2**
L thalamus		3.52	-14	-26	6
L white matter		3.20	-18	-16	-2
**L intracalcarine cortex**	**50**	**4.08**	**-26**	**-78**	**8**
**R cerebellum**	**186**	**4.07**	**38**	**-44**	**-30**
R cerebellum		3.80	28	-52	-24
R cerebellum		3.26	34	-44	-20
**R superior parietal lobe**	**187**	**3.97**	**20**	**-28**	**72**
R postcentral gyrus		3.49	26	-44	58
R postcentral gyrus		3.17	38	-40	64
**L planum temporale**	**245**	**3.94**	**-48**	**-32**	**12**
L superior temporal gyrus		3.32	-56	-28	18
L superior temporal gyrus		3.26	-50	-40	16
**L supramarginal gyrus**	**130**	**3.91**	**-58**	**-38**	**46**
L inferior parietal lobule		3.22	-54	-46	50
L inferior parietal lobule		3.02	-48	-36	48
**R insula**	**159**	**3.87**	**34**	**-12**	**-4**
R insula		3.80	36	-18	6
R insula		3.34	38	-22	-2
**L temporal occipital fusiform cortex**	**65**	**3.80**	**-28**	**-46**	**-18**
L cerebellum		3.23	-26	-56	-12
**R putamen**	**339**	**3.76**	**24**	**10**	**-2**
R white matter		3.74	12	-16	-6
R pallidum		3.60	28	-12	4
**R parietal operculum**	**425**	**3.69**	**54**	**-20**	**20**
R superior temporal gyrus, Te1		3.49	50	-26	14
R superior temporal gyrus		3.47	50	-34	16
**L insula**	**227**	**3.66**	**-42**	**-8**	**-8**
L superior temporal gyrus		3.47	-48	-22	0
L white matter		3.00	-38	-18	-8
**R middle temporal gyrus, temporooccipital**	**53**	**3.51**	**54**	**-54**	**2**
**L medial temporal pole**	**52**	**3.38**	**-24**	**4**	**-42**
L medial temporal pole		2.77	-24	2	-34
**R lateral occipital cortex, inferior**	**56**	**3.35**	**48**	**-76**	**4**
R inferior temporal gyrus		2.82	54	-68	2
R inferior temporal gyrus		2.71	42	-70	2
**L superior parietal lobule**	**70**	**3.26**	**-26**	**-50**	**56**
L superior parietal lobule		2.88	-36	-52	58

L superior parietal lobule		2.73	-30	-56	64
**L inferior occipital gyrus**	**50**	**3.11**	**-52**	**-76**	**2**
SEED R superior temporal gyrus					
**L superior frontal gyrus**	**96**	**4.20**	**-14**	**42**	**44**
L superior frontal gyrus		3.26	-8	38	38
L superior frontal gyrus		2.94	-20	36	48
**R temporal pole**	**56**	**4.06**	**42**	**14**	**-40**
**L middle frontal gyrus**	**193**	**3.89**	**-36**	**14**	**60**
L superior frontal gyrus		3.66	-12	22	52
L middle frontal gyrus		3.55	-20	16	56
**R superior frontal gyrus**	**322**	**3.89**	**6**	**48**	**40**
R superior frontal gyrus		3.77	16	32	54
R superior frontal gyrus		3.71	22	36	46
**R frontal pole**	**92**	**3.86**	**18**	**40**	**-20**
R white matter		3.26	10	46	-26
R middle orbital gyrus		2.74	20	36	-12
**R precuneus**	**215**	**3.75**	**14**	**-48**	**30**
R precuneus		3.62	4	-64	40
L precuneus		3.16	-10	-66	44
**R lateral occipital cortex, superior**	**297**	**3.69**	**48**	**-56**	**28**
R white matter		3.49	34	-66	38
R angular gyrus		3.42	42	-58	34
**L occipital fusiform gyrus**	**73**	**3.67**	**-14**	**-80**	**-18**
L cerebellum		3.22	-16	-86	-26
L cerebellum		3.09	-24	-82	-20
**R occipital fusiform gyrus**	**86**	**3.58**	**10**	**-88**	**-18**
R cerebellum		3.00	16	-82	-22
R cerebellum		2.69	22	-86	-16
**L occipital pole**	**101**	**3.54**	**-8**	**-98**	**2**
**L thalamus, parietal**	**57**	**3.48**	**-18**	**-28**	**0**
**L middle frontal gyrus**	**55**	**3.29**	**-44**	**8**	**48**
**R occipital pole**	**60**	**3.28**	**16**	**-92**	**2**
**R middle frontal gyrus**	**98**	**3.28**	**58**	**12**	**36**
R inferior frontal gyrus, opercularis		2.99	44	12	34
SEED L superior temporal gyrus					
**L precuneus**	**408**	**4.63**	**-6**	**-68**	**40**
L precuneus		4.24	-6	-72	50
R precuneus		3.92	4	-64	38
**L nucleus accumbens**	**109**	**4.01**	**-6**	**24**	**-4**
L caudate nucleus		3.52	-14	22	6
L caudate nucleus		2.67	-8	16	10
**R caudate nucleus**	**163**	**4.00**	**10**	**20**	**6**
R caudate nucleus		3.11	18	22	6
R olfactory cortex		3.03	8	14	-10
**L middle frontal gyrus**	**167**	**3.88**	**-46**	**28**	**36**
L inferior frontal gyrus, triangularis		3.60	-52	14	28
L inferior frontal gyrus, triangularis		3.32	-44	30	28
**L white matter**	**68**	**3.82**	**-18**	**40**	**4**
**L precuneus**	**63**	**3.71**	**2**	**-58**	**16**
L calcarine gyrus		3.38	0	-66	18
**R inferior frontal gyrus, triangularis**	**98**	**3.67**	**44**	**22**	**24**
R inferior frontal gyrus, triangularis		2.85	56	20	24

**L precentral gyrus**	**55**	**3.54**	**-36**	**-2**	**36**
L precentral gyrus		3.07	-44	2	42
**R lateral occipital cortex**	**54**	**3.51**	**34**	**-64**	**28**
**L occipital pole**	**106**	**3.43**	**-10**	**-98**	**-2**
L calcarine gyrus		3.03	-4	-94	-6
L lingual gyrus		3.01	-14	-84	-2
SEED L nucleus accumbens					
**L supramarginal gyrus, posterior**	**1896**	**4.89**	**-54**	**-36**	**30**
L superior temporal gyrus		4.88	-54	-32	22
L supramarginal gyrus		4.34	-58	-26	30
**R insula**	**535**	**4.60**	**40**	**-16**	**-8**
R superior temporal gyrus		4.05	44	-26	4
R insula		3.80	34	-22	8
**R supramarginal gyrus, anterior**	**1218**	**4.45**	**70**	**-26**	**16**
R supramarginal gyrus		4.10	66	-26	30
R rolandic operculum		4.08	54	-30	28
**R temporal pole**	**250**	**4.36**	**54**	**12**	**-8**
R temporal pole		3.95	54	4	0
R temporal pole, Te3		3.08	64	2	-6
**L pallidum**	**53**	**3.69**	**-24**	**-12**	**-2**
**L supplementary motor cortex**	**55**	**3.56**	**-4**	**-12**	**60**
R posterior-medial frontal		2.77	4	-8	58
**R frontal orbital cortex**	**74**	**3.42**	**24**	**24**	**-22**
R superior orbital gyrus		3.30	16	20	-20
**R thalamus, temporal**	**52**	**3.35**	**16**	**-34**	**14**
R thalamus, parietal		2.85	24	-30	14

Supplementary Table 8. **Peak co-ordinates for significant brain regions of the functional network analysis**. The table reports increased functional connectivity during natural versus synthetic and synthetic versus natural identity matching (p<.005 corrected at cluster level k>47, n=25). Seed regions are based on the univariate contrast analysis (Figure 2a, Supplementary Table 5). * indicates significant results at p<.005, k=46. For completeness, we report functional connectivity with the left nucleus accumbens in gray font.

Region	Cluster size	Z value	MNI coordinates		
			x	y	z
SEED R nucleus accumbens					
Contrast: ID_{nat}>ID_{df}					
**R thalamus, parietal**	**163**	**4.24**	**26**	**-24**	**6**
R putamen		3.76	26	4	6
R pallidum		2.91	28	-12	6
**R postcentral gyrus**	**566**	**3.76**	**26**	**-30**	**68**
R white matter		3.62	26	-28	56
R postcentral gyrus		3.59	42	-30	66
**L hippocampus**	**90**	**3.67**	**-36**	**-18**	**-8**
L white matter		3.60	-32	-8	-8
L hippocampus		3.03	-26	-16	-6
**R fusiform gyrus**	**49**	**3.65**	**36**	**-50**	**-16**
**L postcentral gyrus**	**62**	**3.63**	**-58**	**-18**	**52**
L postcentral gyrus		2.89	-62	-20	42
L inferior parietal lobule		2.80	-54	-26	54
Contrast: ID_{df}>ID_{nat}					
**R cerebellum**	**80**	**4.45**	**30**	**-66**	**-30**
**L inferior frontal gyrus, triangularis**	**229**	**3.95**	**-36**	**20**	**26**
L inferior frontal gyrus, triangularis		3.29	-50	22	24
L inferior frontal gyrus, triangularis		2.72	-38	24	16
**L inferior frontal gyrus, orbitalis**	**126**	**3.93**	**-26**	**24**	**-4**
L insula		3.59	-32	16	0
**R precuneus**	**123**	**3.68**	**10**	**-72**	**46**
L cuneus		2.90	2	-76	40
L precuneus		2.84	-2	-66	46
**L occipital cortex, superior**	**109**	**3.54**	**-30**	**-70**	**32**
L white matter		3.24	-32	-54	34
SEED R superior temporal gyrus					
Contrast: ID_{nat}>ID_{df}					
**L brain stem**	**100**	**3.71**	**-10**	**-42**	**-28**
L cerebellum		3.00	-10	-46	-36
**R posterior cingulate cortex**	**59**	**3.64**	**4**	**-50**	**22**
**R lingual gyrus**	**60**	**3.58**	**26**	**-64**	**0**
R fusiform gyrus		3.03	20	-56	-6
**R temporal pole ***	**46**	**3.05**	**30**	**2**	**-36**
Contrast: ID_{df}>ID_{nat}					
	-	-	-	-	-
SEED L superior temporal gyrus					
Contrast: ID_{nat}>ID_{df}					
**R caudate**	**262**	**4.43**	**6**	**18**	**4**
L nucleus accumbens		4.07	-8	26	-4

L caudate nucleus		3.47	-8	18	2
**L superior parietal lobule**	**76**	**3.95**	**-16**	**-58**	**64**
L superior parietal lobule		2.93	-16	-60	56
L superior parietal lobule		2.81	-24	-58	70
**R putamen**	**74**	**3.38**	**26**	**10**	**4**
R pallidum		2.81	24	-6	6
Contrast: ID_{df}>ID_{nat}					
**L supramarginal gyrus**	**185**	**5.03**	**-48**	**-48**	**32**
L supramarginal gyrus		3.61	-46	-52	40
**L inferior temporal gyrus, posterior**	**49**	**3.94**	**-48**	**-26**	**-32**
L inferior temporal gyrus, posterior		2.89	-60	-26	-28
L mid fusiform gyrus		2.71	-42	-34	-30
**L posterior cingulate cortex**	**52**	**3.77**	**-12**	**-46**	**30**
L posterior cingulate cortex		3.14	-8	-38	32
**L precuneus**	**87**	**3.43**	**-6**	**-74**	**28**
L cuneus		3.19	-8	-66	26
L precuneus		3.13	-12	-72	38
**L white matter**	**85**	**3.35**	**-18**	**-22**	**44**
L white matter		2.96	-20	-20	32
SEED L nucleus accumbens					
Contrast: ID_{nat}>ID_{df}					
**L brain stem**	**56**	**4.59**	**-2**	**-22**	**-6**
**L white matter**	**71**	**4.57**	**-32**	**-10**	**26**
L white matter		2.67	-24	-14	26
**L posterior cingulate gyrus**	**64**	**3.86**	**-4**	**-48**	**16**
R posterior cingulate cortex		3.11	4	-44	18
**L insula**	**50**	**3.82**	**-34**	**-10**	**6**
**R planum polare**	**117**	**3.68**	**42**	**-18**	**-8**
R putamen		3.10	34	-10	2
R superior temporal gyrus		3.07	44	-8	-4
**R/L posterior cingulate gyrus**	**55**	**3.49**	**0**	**-40**	**30**
**L lateral occipital gyrus**	**86**	**3.47**	**-38**	**-72**	**26**
**R parahippocampal gyrus, posterior**	**53**	**3.26**	**26**	**-2**	**-28**
**L middle frontal gyrus**	**57**	**3.24**	**-44**	**16**	**48**
L middle frontal gyrus		3.08	-34	18	50
**R planum temporale**	**114**	**3.22**	**66**	**-20**	**8**
R superior temporal gyrus		3.11	50	-32	16
R superior temporal gyrus		2.89	60	-24	14
Contrast: ID_{df}>ID_{nat}					
**L inferior frontal gyrus, triangularis**	**82**	**3.68**	**-36**	**18**	**26**
**L brain stem**	**80**	**3.66**	**-6**	**-34**	**-6**
L brain stem		3.41	-12	-32	-14
L brain stem		2.75	-6	-40	-18
**R precuneus**	**166**	**3.56**	**14**	**-70**	**44**
R precuneus		3.50	4	-68	44
R cuneus		3.16	18	-62	44
**L superior parietal lobe**	**59**	**3.47**	**-44**	**-44**	**34**
L inferior parietal lobule		3.00	-38	-46	46
L white matter		2.85	-32	-54	36

Supplementary Table 9. Within-class classification accuracies of the multivariate decoding analysis. We report mean classification accuracies, standard deviations as revealed by confusion matrices, and t-statistics. Bold font indicates significant above-chance performance (25% chance level) based on one-sample t-tests (*p*<.05, Bonferroni corrected for the number of AC subregions, n=24). For completeness, we report decoding analysis within the left NAcc in gray font. Abbreviations: NAcc: nucleus accumbens, AC: auditory cortex, IFG, tri: inferior frontal gyrus, pars triangularis, TP: temporal pole.

True = predicted class	ID_{nat}		ID_{df}		$SPEECH_{nat}$		$SPEECH_{df}$	
	Mean % (SD)	t-statistic	Mean % (SD)	t-statistic	Mean % (SD)	t-statistic	Mean % (SD)	t-statistic
**Right NAcc**	**33.33 (12.57)**	t=3.25 *p*=.002	27.50 (8.34)	t=1.47 *p*=.08	24.17 (14.04)	t=-0.29 *p*=.61	23.33 (10.90)	t=-0.75 *p*=.77
**Left Nacc**	**34.17 (10.60)**	t=4.24 *p*<.001	26.25 (13.04)	t=0.47 *p*=.32	**30.42 (11.79)**	t=2.25 *p*=.02	23.33 (8.43)	t=-0.14 *p*=.56
**Right AC**								
TE1	**38.33 (14.19)**	t=4.60 *p*<.0001	**33.54 (10.98)**	t=3.81 *p*<.001	**34.17 (10.70)**	t=4.20 *p*<.001	26.04 (10.53)	t=0.49 *p*=.32
TE2.1	**36.88 (12.41)**	t=4.69 *p*<.0001	**31.04 (7.51)**	t=3.94 *p*<.001	**35.42 (13.18)**	t=3.87 *p*<.001	31.46 (12.55)	t=2.52 *p*=.01
TE2.2	**38.33 (13.49)**	t=4.84 *p*<.0001	**33.33 (11.29)**	t=3.62 *p*=.001	**35.21 (10.48)**	t=4.77 *p*<.0001	**34.38 (11.06)**	t=4.15 *p*<.001
TEI	**39.38 (16.11)**	t=4.37 *p*<.001	**34.17 (11.95)**	t=3.76 *p*<.001	**35.00 (13.35)**	t=3.67 *p*<.001	27.29 (12.16)	t=0.92 *p*=.18
TE3	**40.83 (13.49)**	t=5.75 *p*<.0001	**37.92 (11.88)**	t=5.33 *p*<.0001	**37.08 (12.24)**	t=4.84 *p*<.0001	**36.67 (13.73)**	t=4.16 *p*<.001
STS	**42.71 (12.07)**	t=7.19 *p*<.0001	31.67 (13.49)	t=2.42 *p*=.012	**38.13 (13.17)**	t=4.88 *p*<.0001	**33.33 (12.38)**	t=3.27 *p*=.002
**Left AC**								
TE1	**38.96 (15.67)**	t=4.36 *p*<.001	**33.33 (12.40)**	t=3.29 *p*=.002	**33.96 (13.10)**	t=3.35 *p*=.001	29.17 (13.41)	t=1.52 *p*=.07
TE2.1	**34.79 (12.81)**	t=3.75 *p*<.001	**33.33 (12.67)**	t=3.23 *p*=.002	27.71 (13.91)	t=0.95 *p*=.18	**32.50 (13.75)**	t=2.67 *p*=.007
TE2.2	**38.75 (13.93)**	t=4.84 *p*<.0001	**34.79 (13.79)**	t=3.48 *p*=.001	**34.58 (12.15)**	t=3.86 *p*<.001	**31.25 (11.45)**	t=2.68 *p*=.007
TEI	**45.00 (15.25)**	t=6.42 *p*<.0001	**35.21 (14.03)**	t=3.57 *p*<.001	**35.42 (14.14)**	t=3.61 *p*<.001	30.63 (13.62)	t=2.02 *p*=.03
TE3	**44.79 (13.15)**	t=7.38 *p*<.0001	**41.67 (11.48)**	t=7.11 *p*<.0001	**37.29 (10.32)**	t=5.84 *p*<.0001	**34.17 (12.57)**	t=3.57 *p*<.001
STS	**46.88 (13.25)**	t=8.09 *p*<.0001	**33.54 (12.02)**	t=3.48 *p*=.001	32.29 (14.74)	t=2.42 *p*=.01	**32.29 (10.53)**	t=3.39 *p*=.001
**Right IFG, tri**	**47.71 (13.35)**	t=8.33 *p*<.0001	**41.46 (11.47)**	t=7.03 *p*<.0001	**47.92 (20.37)**	t=5.51 *p*<.0001	**37.29 (11.42)**	t=5.27 *p*<.0001
**Left IFG, tri**	**50.00 (13.51)**	t=9.06 *p*<.0001	**37.71 (13.35)**	t=4.66 *p*<.0001	**41.46 (15.07)**	t=5.35 *p*<.0001	**41.25 (13.04)**	t=6.10 *p*<.0001
**Right TP**	**45.83 (16.53)**	t=6.18 *p*<.0001	**36.67 (12.48)**	t=4.58 *p*<.0001	**38.33 (12.74)**	t=5.13 *p*<.0001	30.00 (15.39)	t=1.59 *p*=.06
**Left TP**	**42.50 (15.81)**	t=5.42 *p*<.0001	**34.79 (11.84)**	t=4.05 *p*<.001	**38.33 (12.74)**	t=5.13 *p*<.0001	**30.83 (12.57)**	t=2.27 *p*=.02

Supplementary Table 10. Between-class confusion for the identity classes of the multivariate decoding analysis. We report mean confusion values, standard deviations as revealed by confusion matrices, and t-statistics. Bold font indicates significant above-chance confusion (25% chance level) based on one-sample t-tests (p<.05, Bonferroni corrected for the number of AC subregions, n=24). For completeness, we report decoding analysis within the left NAcc in gray font. Abbreviations: NAcc: nucleus accumbens, AC: auditory cortex, IFG, tri: inferior frontal gyrus, pars triangularis, TP: temporal pole.

True and predicted class	**ID_{nat} & ID_{df}**		**ID_{df} & ID_{nat}**	
	Mean % (SD)	t-statistic	Mean % (SD)	t-statistic
**Right NAcc**	**31.46 (10.37)**	t=3.05, *p*=.003	26.88 (11.21)	t=0.82, *p*=.21
**Left NAcc**	**34.79 (10.16)**	t=4.72, *p*<.0001	24.79 (7.29)	t=-0.14, *p*=.56
**Right AC**<br>TE1	**34.58 (10.83)**	t=4.34, *p*<.001	27.08 (11.12)	t=0.92, *p*=.18
TE2.1	**34.37 (14.71)**	t=4.68, *p*<.0001	26.46 (9.82)	t=0.49, *p*=.32
TE2.2	**33.96 (15.81)**	t=2.78, *p*=.005	26.88 (11.11)	t=0.83, *p*=.21
TEI	**39.17 (14.04)**	t=4.94, *p*<.0001	27.08 (12.76)	t=0.80, *p*=.22
TE3	**34.38 (14.17)**	t=3.24, *p*=.002	26.46 (12.20)	t=0.59, *p*=.28
STS	**39.17 (12.57)**	t=5.52, *p*<.0001	26.67 (13.32)	t=0.61, *p*=.27
**Left AC**<br>TE1	**31.46 (9.61)**	t=3.29, *p*=.002	22.29 (10.93)	t=-1.21, *p*=.88
TE2.1	30.63 (12.45)	t=2.21, *p*=.02	23.96 (12.16)	t=-0.42, *p*=.66
TE2.2	**32.71 (12.42)**	t=3.04, *p*=.003	23.54 (10.05)	t=-0.71, *p*=.76
TEI	**32.50 (12.34)**	t=2.98, *p*=.003	20.83 (10.29)	t=-1.98, *p*=.97
TE3	**33.54 (11.75)**	t=3.56, *p*<.001	25.83 (12.31)	t=0.33, *p*=.37
STS	**39.17 (11.20)**	t=6.20, *p*<.0001	24.79 (10.05)	t=-0.10, *p*=.54
**Right IFG, tri**	**45.21 (10.48)**	t=9.45, *p*<0001	**34.17 (12.22)**	t=3.68, *p*<.001
**Left IFG, tri**	**44.38 (12.80)**	t=7.42, *p*<.0001	**30.21 (10.58)**	t=2.41, *p*=.01
**Right TP**	**36.46 (13.79)**	t=4.07, *p*<.001	23.13 (9.42)	t=-0.98, *p*=.83
**Left TP**	**39.17 (9.17)**	t=7.57, *p*<.0001	27.08 (8.96)	t=1.14, *p*=.13

Supplementary Table 11. Social perception ratings of natural and deepfake sounds and correlation matrix between rating dimensions. a) Mean and standard deviation of perceptual ratings on each social dimension for all sounds and for the natural and deepfake sounds separately. b) Correlation coefficients of the correlation matrix. $n_{participants}$=25, $n_{observations}$=14394.

**a**	**All sounds** Mean (SD)	**Natural sounds** Mean (SD)	**Deepfake sounds** Mean (SD)
**Naturalness ratings**	3.23 (1.63)	4.55 (0.88)	1.89 (1.00)
**Likability ratings**	3.03 (1.37)	3.91 (1.07)	2.13 (1.02)
**Trustworthiness ratings**	2.87 (1.40)	3.75 (1.14)	1.98 (1.03)
**b**	**Naturalness** (all sounds)	**Likability** (all sounds)	**Trustworthiness** (all sounds)
**Naturalness** (all sounds)	1.00	0.81	0.78
**Likability** (all sounds)		1.00	0.91
**Trustworthiness** (all sounds)			1.00

Supplementary Table 12. Social perception of deepfake and natural sounds. Model outputs for the CLMMs assessing social perception rating differences for the natural and deepfake sounds. For factors, reference level is shown in brackets. We ran separate models for each rating dimension. $n_{participants}$=25, $n_{observations}$=20016.

**Factors predicting social perception**	**Odds ratio**	**CI**	***p* value**
**Naturalness ratings**			
Sound condition (SC) [deepfake]	0.00	0.00;0.01	<.001
Variance of random slopes: sc|participant=4.68, sc|speaker=0.23, Conditional R^2 =0.784			
**Likability ratings**			
Sound condition [deepfake]	0.02	0.01;0.04	<.001
Variance of random slopes: sc|participant=2.30, sc|speaker=0.56, Conditional R^2 =0.673			
**Trustworthiness ratings**			
Sound condition [deepfake]	0.01	0.00;0.04	<.001
Variance of random slopes: sc|participant=3.44, sc|speaker=0.70, Conditional R^2 =0.715			

Supplementary Table 13. Social relevance of neural response in the NAcc and AC during the fMRI identity task. Model outputs for the linear regression models assessing effects of perceptual social ratings for the neural response difference between the natural and deepfake identity task (ID_{nat}-ID_{df}) for the respective ROIs; n=25.

**Factors predicting neural difference during identity task ID_{nat}-ID_{df}**	**Estimate**	**CI**	***p* value**
**Right AC**			
Naturalness ratings NAT_{nat}-NAT_{df}	-0.41	-0.81;-0.01	**.044**
Likability ratings LIK_{nat}-LIK_{df}	-0.34	-0.78;0.11	.130
Trustworthiness ratings $TRUST_{nat}$-$TRUST_{df}$	-0.16	-0.53;0.21	.377
**Left AC**			
Naturalness ratings NAT_{nat}-NAT_{df}	-0.46	-0.90;-0.02	**.041**
Likability ratings LIK_{nat}-LIK_{df}	-0.59	-1.04;-0.15	**.011**
Trustworthiness ratings $TRUST_{nat}$-$TRUST_{df}$	-0.33	-0.86;0.20	.206
**Right NAcc**			
Naturalness ratings NAT_{nat}-NAT_{df}	0.16	-0.21;0.53	.386
Likability ratings LIK_{nat}-LIK_{df}	-0.17	-0.57;0.22	.374
Trustworthiness ratings $TRUST_{nat}$-$TRUST_{df}$	-0.17	-0.49;0.14	.273

Supplementary Table 14. Social relevance of neural response in the AC during the fMRI speech task. Model outputs for the linear regression models assessing effects of perceptual social ratings for the neural response difference between the natural and deepfake speech task (ID_{nat}-ID_{df}) for the respective ROIs; n=25.

**Factors predicting neural difference during speech task $SPEECH_{nat}$-$SPEECH_{df}$**	**Estimate**	**CI**	***p* value**
**Right AC**			
Naturalness ratings NAT_{nat}-NAT_{df}	-0.05	-0.50;0.39	.801
Likability ratings LIK_{nat}-LIK_{df}	-0.05	-0.53;0.42	.813
Trustworthiness ratings $TRUST_{nat}$-$TRUST_{df}$	-0.07	-0.45;0.32	.716
**Left AC**			
Naturalness ratings NAT_{nat}-NAT_{df}	-0.13	-0.76;0.50	.680
Likability ratings LIK_{nat}-LIK_{df}	-0.27	-0.93;0.40	.416
Trustworthiness ratings $TRUST_{nat}$-$TRUST_{df}$	-0.33	-0.86;0.20	.206

References

1. G. Dutilh, D. van Ravenzwaaij, S. Nieuwenhuis, H. L. J. van der Maas, B. U. Forstmann, E.-J. Wagenmakers, How to measure post-error slowing: A confound and a simple solution. *J. Math. Psychol.* **56**, 208–216 (2012).
